# Immediate Effects of Walker-Assisted Gait Training at Higher Training Speeds Comparing Conditions With and Without Body Weight Support After Stroke: A Pilot Crossover Study

**DOI:** 10.7759/cureus.102772

**Published:** 2026-02-01

**Authors:** Hiroo Koshisaki, Shota Nagai

**Affiliations:** 1 Department of Rehabilitation, Nanto Municipal Hospital, Nanto, JPN; 2 Graduate School of Comprehensive Rehabilitation, Kinjo University, Hakusan, JPN

**Keywords:** body weight support, immediate effect, stroke, training speed, walker

## Abstract

Background: Body weight-supported treadmill training (BWSTT) permits patients with stroke to practice walking at higher speeds under safe conditions but is limited by cost and accessibility. A suspended body weight-supported (BWS) walker has been developed as a practical alternative, enabling higher training speeds during walker-assisted gait training. However, it is unclear whether high-speed BWS walker-assisted gait training leads to immediate improvements in gait performance and how training speed and body weight support contribute to these effects.

Methods: Twenty patients with chronic stroke participated in a crossover study. Each participant underwent two gait training sessions using the same walker device: one without BWS and one with BWS activated. Gait performance was assessed before and after each session using the 10 m walk test. Training speed during each session was recorded to evaluate its relationship with pre-to-post changes in gait performance.

Results: Training with BWS enabled higher training speeds and resulted in significant improvements in gait speed and affected side step time, with a significantly greater increase in gait speed than that during training without BWS. Of the participants, 70% demonstrated increased training speed accompanied by immediate improvements in gait speed during BWS walker-assisted training. Improvements in gait speed were associated with increased stride length and reduced affected side step time.

Conclusion: Walker-assisted gait training that enables higher training speeds, particularly when combined with BWS, may contribute to immediate improvements in gait speed in patients with stroke. Reductions in affected side step time may represent one mechanism underlying this training speed-related effect.

## Introduction

Stroke patients often experience reduced gait speed due to functional impairments such as hemiplegia [1]. Improving gait speed is a critical rehabilitation goal, as it is directly associated with activities of daily living [2]. The efficacy of suspended body weight-supported (BWS) treadmill training (BWSTT) in enhancing gait speed in stroke patients has been well documented [3-5]. One of the major advantages of BWSTT is that the use of a safety harness allows precise control of walking speed while minimizing the risk of falls. This enables patients to practice walking at speeds higher than their self-selected walking speed under safe conditions. Gait training performed at higher walking speeds is associated with improvements in gait speed and spatiotemporal gait parameters in patients with stroke [6,7]. These speed-dependent training effects are considered an important mechanism underlying gait improvement. However, suspension-based BWS devices such as those used for BWSTT have notable limitations, including high cost, lack of portability, and restricted availability to specialized facilities. To address these drawbacks, a suspended BWS walker has been developed [8].

To prevent falls and make weight unloading during gait training easier, the BWS walker uses a harness fastened to the walker to support body weight. Recent studies on BWS walkers have shown that the use of a suspension system reduces the fear of falling and enables patients with stroke to walk at higher training speeds than those during usual walking [9]. Importantly, both walker-assisted walking without body weight support and walking with body weight support allowed patients to achieve faster walking speeds, suggesting that the presence of a safety suspension itself plays a key role in facilitating higher training speeds by enhancing postural confidence.

Although it has become clear that walker-assisted gait training enables patients to walk at higher training speeds under safe conditions, it remains unclear whether such high-speed gait training translates into improvements in actual walking performance. In addition, the relative contributions of training speed and body weight support to immediate gait improvements have not been fully elucidated.

In this study, we aimed to investigate the immediate effects of walker-assisted gait training at higher training speeds by comparing conditions with and without body weight support in patients with stroke.

## Materials and methods

Study design and experimental protocol

**Figure 1:**
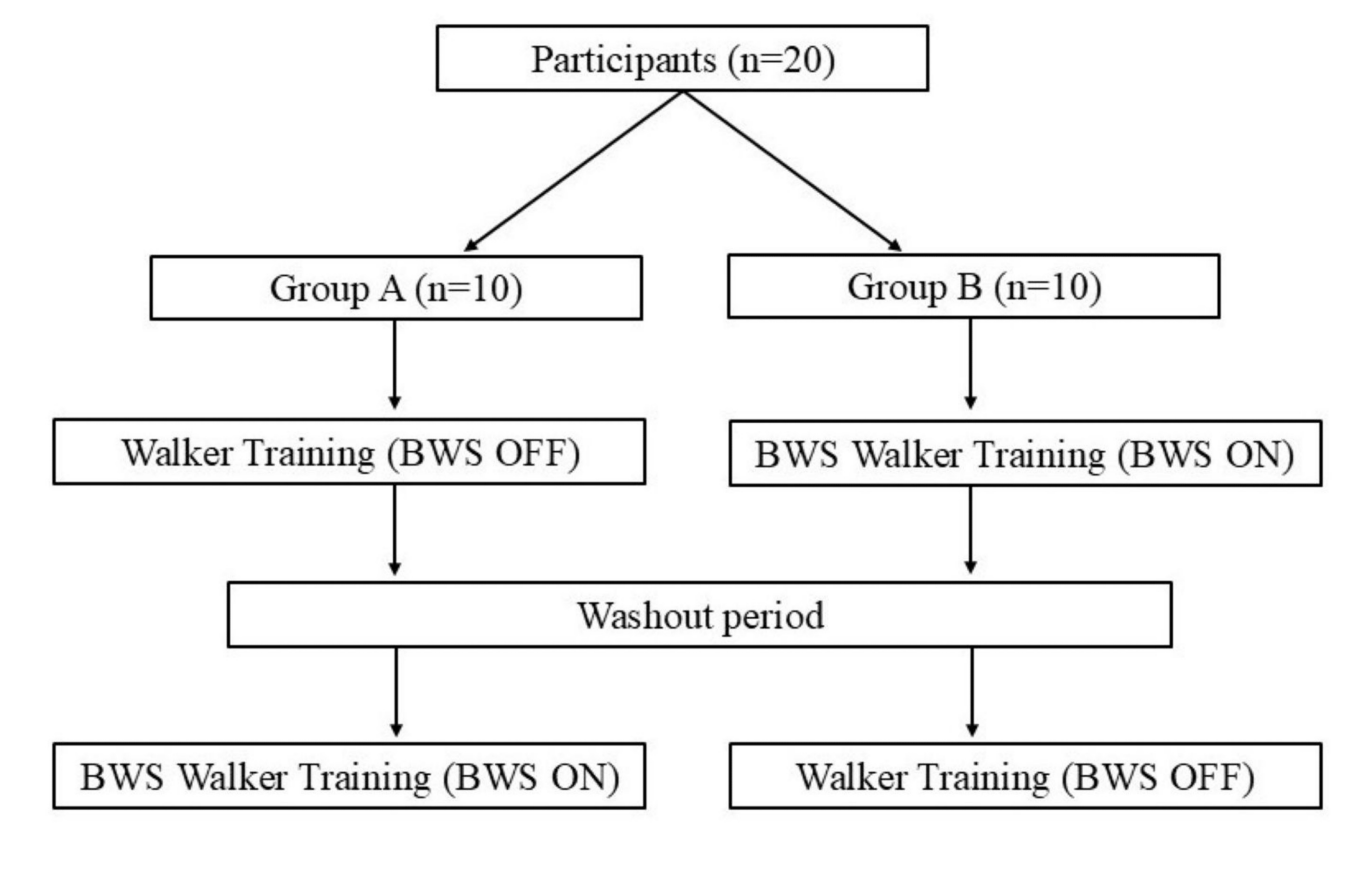
Study design of the open-label crossover trial. BWS: body weight-supported.

The immediate effects of gait training were assessed by comparing gait performance using the same suspended walker device with BWS activated (BWS walker condition) or without BWS (walker condition). The study design employed an open-label crossover design (Figure 1).

Participants were randomly assigned to one of two groups: group A first underwent walker gait training, followed by a one-week washout period, and then BWS walker gait training. Group B first underwent BWS walker gait training, followed by a one-week washout period, and then walker gait training. Walker gait training was performed without body weight support, whereas BWS walker gait training included 25% body weight support. Each training session lasted 10 minutes, including rest periods. The intervention was conducted under therapist supervision, and no falls, accidents, or other adverse events occurred during the study period. Pre- and post-training gait performance was assessed using a 10-meter walk test. The differences in gait parameters before and after each training session were analyzed, and the magnitude of change was compared between walker and BWS walker gait training. Additionally, gait speed during each training session was calculated for both conditions.

Participants

Twenty stroke patients (mean age: 74.6 ± 7.9 years) who were either hospitalized or attending rehabilitation were included in this study (Table 1).

**Table 1:** Clinical characteristics of the participants. FAC: functional ambulation categories; FMA: Fugl-Meyer Assessment.

Variables	Values
Age (years), mean±SD	74.6±7.9
Sex, n	
Male	13
Female	7
Affected side, n	
Right	8
Left	12
Diagnosis, n	
Cerebral infarction	10
Cerebral hemorrhage	10
Time since stroke (months), mean±SD	71.5±85.2
Walking aid (usual walking), n	
Yes	18
No	2
Orthosis, n	
Yes	11
No	9
Functional ambulation category, median (IQR)	3 (2–4)
Lower extremity FMA, mean±SD	20.2±6.7

The inclusion criteria were as follows: hemiplegia with a Fugl-Meyer Assessment lower limb score of ≤33, a functional ambulation category score of ≥2, the ability to walk at least 16 m, and a post-stroke duration of ≥3 months. The exclusion criteria included acute stroke (<3 months after onset), severe dementia, or higher brain dysfunction that impaired comprehension or execution of the study protocol. All participants received verbal and written explanations regarding the study objectives, methodology, potential risks, and personal data protection. Written informed consent was obtained before participation. The study was approved by the Nanto Municipal Hospital Medical Ethics Committee (Approval No. 709) and registered in the clinical trials registry (UMIN000054671).

Equipment

**Figure 2:**
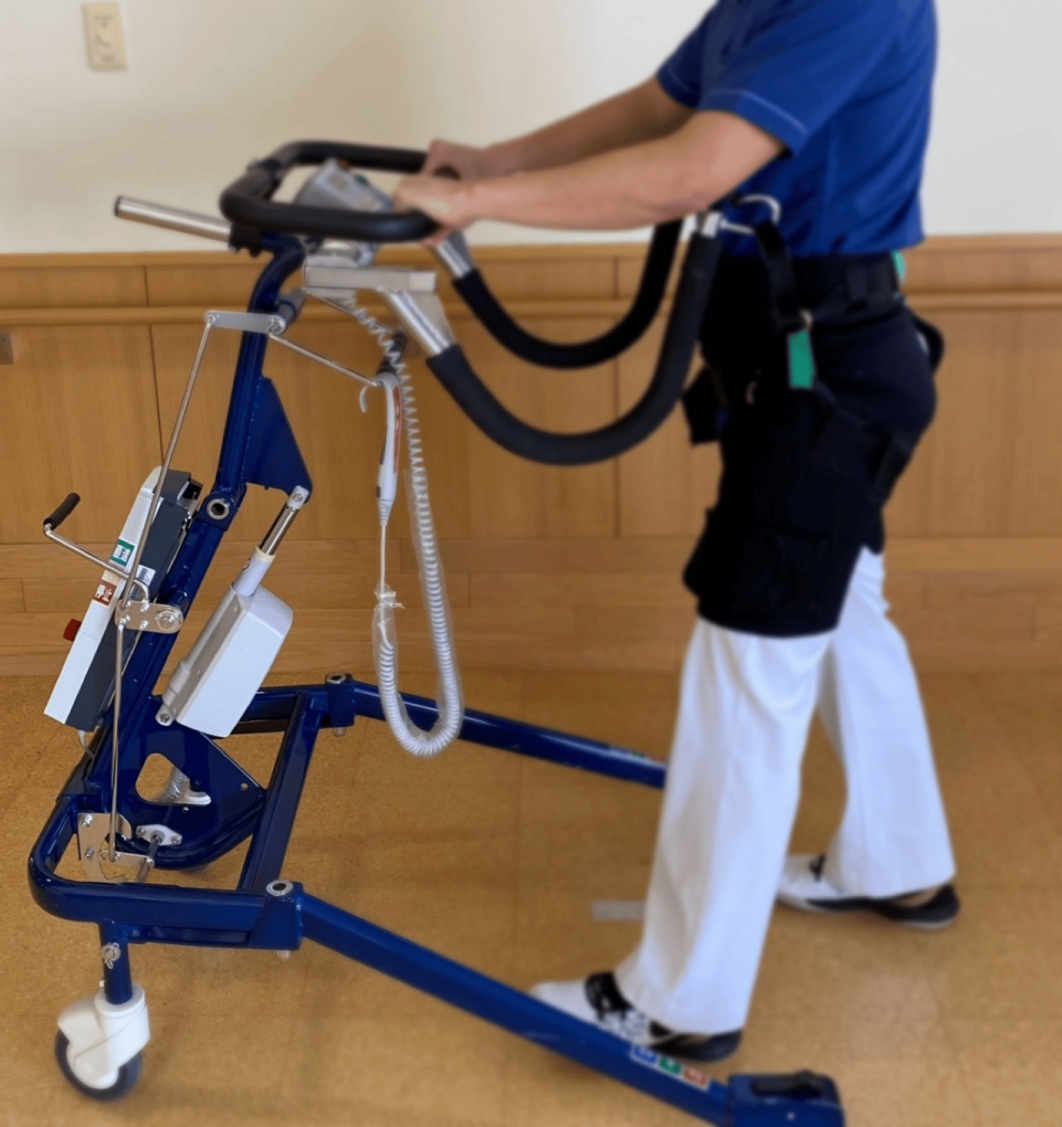
Walker used in the study. The same device was used for both the walker and body weight-supported (BWS) conditions.

The Relief Walking Lift POPO (Moritoh Corporation, Ichinomiya, Japan) (Figure 2) was used as the walker and the BWS walker in this study.

This walker-type device prevents falls and reduces lower limb load by suspending the body using a harness and a belt attached to the lower trunk. The device allows unloading of up to 40 kg.

For gait analysis, an acceleration sensor (Gait-kun MG-M1110-HW, LSI Medience Corporation, Tokyo, Japan) was used, with data analyzed on a dedicated system (Gait View MG-M1110-PC). Before measurement, the accelerometer was calibrated (zero-point correction) for each participant. The sensor was secured with a specialized belt in a pocket over the spinous process of the third lumbar vertebra [10]. Gait assessment was conducted using a 10-meter walk test with the accelerometer attached. A remote control switch was used to mark the start and end of the 10-m walk. Data were recorded at a sampling frequency of 100 Hz. Heel contact was identified based on the peak of the anterior-posterior acceleration waveform, following established methods [11]. Using these data, step time for the affected and unaffected sides was determined, and gait symmetry was calculated as the symmetry ratio (affected side step time/unaffected side step time) [12]. Gait speed (m/s) was determined by dividing the walking distance (10 m) by the walking time, which was identified using foot-switch signals marking the start and end of the walking section. Stride length (cm) was calculated by dividing the walking distance by the number of strides detected during the 10 m walk. Because these gait parameters were derived using different measurement approaches and temporal resolutions, they were analyzed independently.

Statistical analysis

First, a paired t-test was used to compare baseline gait speed and training speed for both the walker and BWS walker conditions. Next, a paired t-test was conducted to evaluate differences in gait parameters before and after training for both conditions. Second, a paired t-test was used to compare the magnitude of change in gait parameters between walker and BWS walker training. Finally, Pearson’s correlation coefficients were calculated between post-intervention gait speed and the following gait parameters: stride length, affected side step time, unaffected side step time, and gait symmetry ratio. This analysis aimed to identify which gait variables were most strongly associated with improvements in walking speed after the intervention. A p < 0.05 was considered statistically significant. Data analysis was performed using JMP version 14.2 software (SAS Institute Inc., Cary, NC, USA).

## Results

Training speed

During walker-assisted gait training, the pre-training gait speed was 0.39 ± 0.25 m/s, while the training speed with the walker increased to 0.48 ± 0.24 m/s, demonstrating a significant improvement (p < 0.05). Among the participants, 60% (12 of 20) exhibited an immediate increase in walking speed when training speed was increased concomitantly. During BWS walker-assisted gait training, the pre-training gait speed was 0.39 ± 0.24 m/s, and the training speed with the BWS walker increased to 0.50 ± 0.25 m/s (p < 0.05). Notably, 70% (14 of 20) of participants demonstrated an immediate improvement in gait speed as training speed increased concomitantly. These findings indicate that for many participants, an increase in training speed was associated with an immediate enhancement in gait performance.

Immediate effects and magnitude of change

Walker-assisted gait training resulted in a significant improvement in both affected and unaffected side step time (p < 0.05) (Table 2). BWS walker-assisted gait training led to a significant improvement in both gait speed and affected side step time (p < 0.05) (Table 2). Furthermore, the increase in gait speed following BWS walker-assisted training was significantly greater than that observed with standard walker-assisted training (p < 0.05) (Table 2). Although stride length and gait symmetry exhibited a trend toward improvement during BWS walker-assisted gait training, no significant differences were observed.

**Table 2:** Pre- and post-intervention values and between-group comparisons of change. Values are presented as mean ± SD. Within-condition p-values were calculated using paired t-tests. Between-condition p-values represent comparisons of pre–post changes between walker and BWS walker conditions. * P < 0.05.

Outcome	Condition	Pre	Post	Within condition p-value	Between difference condition p-value
Gait speed (m/s)	Walker	0.39±0.25	0.41±0.27	0.1131	
	BWS walker	0.39±0.24	0.43±0.27	0.0101*	0.0409*
Stride length (cm)	Walker	57.2±26.3	56.8±27.2	0.3629	
	BWS walker	56.5±25.9	58.4±26.8	0.0704	0.0732
Affected side step time (sec)	Walker	1.11±0.49	1.06±0.46	0.0260*	
	BWS walker	1.07±0.44	1.03±0.42	0.0061*	0.9909
Unaffected side step time (sec)	Walker	0.59±0.13	0.57±0.12	0.0026*	
	BWS walker	0.58±0.13	0.57±0.13	0.6797	0.0192*
Symmetry ratio (affected side/unaffected side)	Walker	1.93±0.99	1.93±0.96	0.8832	
	BWS walker	1.96±1.01	1.89±1.00	0.0953	0.1344

Correlation between post-intervention gait speed and other gait parameters (BWS walker group)

Significant correlations were observed between gait speed and stride length, affected side step time, and symmetry ratio (p < 0.05) (Table 3). Thus, as walking speed increased, stride length increased, the affected side step time shortened, and the symmetry ratio was improved. No correlation was found between unaffected side step time and gait speed.

**Table 3:** Correlations between post-intervention gait speed and other gait parameters (BWS walker condition). * P < 0.05.

Outcome	Pearson's r	p-value
Stride length	0.9447	<0.001*
Affected side step time	-0.6558	0.0017*
Unaffected side step time	-0.1244	0.6012
Symmetry ratio	-0.5354	0.0150*

## Discussion

In stroke patients, gait training with both the standard walker and the BWS walker resulted in increased training speed, which tended to produce an immediate improvement in gait speed. Notably, gait training with the BWS walker led to a significantly greater improvement in gait speed compared to training with the standard walker.

Regarding the immediate effects of training at an increased gait speed, more than 60% of participants in both groups exhibited an immediate improvement in walking speed when training at a pace faster than their usual walking speed. Several studies have reported that stroke patients can enhance their gait speed by practicing at speeds exceeding their normal walking pace on a treadmill [6,7]. In this study, the standard walker enabled high-speed gait training, and this practice effect appeared to contribute to gait speed improvement.

However, a significant difference was observed in the magnitude of gait speed improvement between the two training methods, with BWS walker training leading to a greater immediate increase in walking speed. BWS-assisted walking has been shown to reduce increases in heart rate and oxygen consumption [13]. In general, stroke patients experience a decline in gait speed after two minutes of walking, which contributes to gait asymmetry [14]. In this study, participants underwent 10 minutes of gait training, including breaks, and their walking ability was evaluated immediately after training. Fatigue may have influenced the results, as partial body weight support during BWS walker training likely reduced fatigue compared to standard walker training, thereby contributing to a more pronounced improvement in gait speed.

Although the intervention resulted in statistically significant improvements in gait speed, the immediate effects observed after a single session were relatively modest. To further investigate the underlying factors associated with gait improvement, we performed an exploratory correlation analysis using post-intervention data. The results demonstrated that gait speed was moderately to strongly correlated with stride length, affected side step time, and step time symmetry. These findings are consistent with previous research indicating that gait symmetry and stride length are critical determinants of walking ability in individuals with stroke [12,15]. It is plausible that improvements in step timing on the paretic side enhanced temporal symmetry, which in turn contributed to increased walking speed. Additionally, a longer stride length may reflect improved propulsion capacity or better interlimb coordination, both of which have been associated with greater gait efficiency [16,17]. Interestingly, unaffected side step time did not show a significant association with gait speed, suggesting that this limb may function in a compensatory and more stable manner, and is thus less sensitive to intervention-driven changes. These findings highlight the importance of targeting the temporal characteristics of the affected side limb in BWS walker gait training aimed at improving gait performance.

This study has several limitations. As the sample size was limited to 20 participants, analyses according to participant characteristics could not be conducted. The immediate effect of a single gait training session resulted in only minor changes in parameters, i.e., the improvement in gait speed was insufficient. Tilson et al. identified a minimal clinically important difference (MCID) of 0.16 m/s over 40 days in patients with subacute stroke [18], whereas Perera et al. suggested that an appropriate gait speed improvement in older adults was approximately 0.1 m/s [19]. The present study differs in that it included patients in the chronic phase (≥3 months post-stroke) and focused on the immediate effects of a single gait training session. The observed change in gait speed was approximately 0.04 m/s. Future research should investigate whether continued use of the BWS walker could lead to gait speed improvements that exceed the MCID over the mid- to long-term.

## Conclusions

The findings of this study indicate that combining walker-assisted walking with partial BWS enables gait training at higher gait speeds, leading to an immediate improvement in gait speed. The improvement in gait speed with the BWS walker appeared to be primarily associated with a reduction in the affected side step time. The improvement in gait speed achieved through high-speed gait training using a BWS walker suggests that this approach may produce effects similar to those observed with BWSTT, while offering greater flexibility in routine clinical settings. Future research should investigate the medium- and long-term effects of BWS walker training to determine whether these benefits are sustained over time.
